# Transcriptomic and Epitranscriptomic Landscape of Integrated HTLV-1 in MT2 Cells

**DOI:** 10.3390/v18010057

**Published:** 2025-12-30

**Authors:** Shuanglong Wei, Bohan Zhang, Jingwan Han, Hanping Li, Yongjian Liu, Lei Jia, Jingyun Li, Xiaotian Huang, Lin Li

**Affiliations:** 1 School of Public Health and Health Management, Gannan Medical University, Ganzhou 341000, China; a384305818@163.com; 2 Department of Virology, Beijing Institute of Microbiology and Epidemiology, Beijing 100071, China; zbhforjob@163.com (B.Z.); hanjingwan@outlook.com (J.H.); hanpingline@163.com (H.L.); yongjian325@sina.com (Y.L.); 15001193408@163.com (L.J.); lijyjk@163.com (J.L.)

**Keywords:** HTLV-1, DRS, splice, modifications, polyadenylation

## Abstract

Human T-lymphotropic virus type 1 (HTLV-1), the first human retrovirus identified, is linked to adult T-cell leukemia and HTLV-1-associated myelopathy/tropical spastic paraparesis. However, its post-transcriptional regulation remains poorly understood. Here, we used Oxford Nanopore direct RNA sequencing to profile the HTLV-1 transcriptome and epitranscriptome in MT2 cells. We identified 23 transcript isoforms, encompassing canonical and novel splice variants. Polyadenylation analysis revealed a predominant poly(A) tail length of around 50–100 nucleotides with transcript-specific variations. Distinct RNA modifications, including pseudouridine, N^6^-methyladenosine, and 5-methylcytidine, were enriched near the 3′ end and varied among transcript classes, with generally lower modification ratios in viral transcripts. These findings provide a more comprehensive map of HTLV-1 RNA splicing, polyadenylation, and modifications in MT2 cells, offering new insights into viral gene regulation and pathogenic mechanisms.

## 1. Introduction

Numerous studies have verified that the replication of viruses is strictly regulated by the host cells. Post-transcriptional RNA processing, including alternative splicing, 3′ polyadenylation, and epitranscriptomic modifications, is an important regulatory mechanism towards viral replication [1,2,3,4]. Human T-lymphotropic virus type 1 (HTLV-1) was the first human retrovirus to be identified in 1980 and is the most pathogenic and widespread member of the HTLV family; studies have confirmed its close association with adult T-cell leukemia (ATL) and HTLV-1-associated myelopathy/tropical spastic paraparesis (HAM/TSP) [5,6,7,8]. However, the complex post-transcriptional processing modifications and comprehensive transcriptional mechanisms of HTLV-1 remain unclear. Therefore, comprehensively elucidating the post-transcriptional processing and modification status of the virus within host cells is of great significance for studying the replication and pathogenicity of HTLV-1.

RNA alternative splicing is effectively involved in regulating gene expression, essential for cellular proliferation, differentiation, and survival [9]. Viruses, especially retroviruses, exhibit complex splicing patterns as well [1,10,11]. HTLV-1 generates multiple transcripts through alternative splicing, including gag-pol-pro, env, tax, rex, and the antisense transcript HBZ [12]. These transcripts enhance immune evasion and T-cell clonal expansion, thereby facilitating progression from asymptomatic infection to ATL [13]. Although several transcript types have been reported previously [14,15], current knowledge of HTLV-1 splicing remains fragmented and incomplete. Previous studies have mostly relied on short-read sequencing or targeted RT-PCR [16,17], which cannot resolve full-length splice isoforms or accurately define donor–acceptor combinations. As a result, the global organization of HTLV-1 splice junctions, the full repertoire of isoform diversity, and the relative abundances of individual splice products have never been systematically characterized. A comprehensive, unbiased, isoform-resolved analysis of HTLV-1 alternative splicing is therefore still lacking.

Besides alternative splicing, 3′ end polyadenylation is another critical post-transcriptional event that affects mRNA stability and translation efficiency [18,19]. The length of poly(A) tail directly impacts the translation efficiency of mRNA [20]. In viruses, polyadenylation plays a critical role in regulating mRNA translational efficiency and stability, and the inflammation it mediates constitutes a key pathological basis for various viral infections [21,22,23]. Up to now, research on the polyadenylation level of retroviral RNA has primarily focused on HIV, while studies on the poly(A) tail of HTLV transcripts remain unexplored [24,25]. No prior study has examined the distribution of poly(A) tail lengths across different HTLV-1 transcripts, nor the potential regulatory roles of poly(A) variation in viral RNA stability or translation. Consequently, how polyadenylation contributes to HTLV-1 RNA gene expression remains essentially unknown.

RNA epitranscriptomics constitute essential post-transcriptional regulatory mechanisms influencing mRNA stability, splicing, localization, and translation efficiency [26]. Although RNA modifications have been extensively studied in retroviruses, most research has focused on HIV. Multiple types of RNA modifications facilitate HIV-1 replication and infection by modulating viral RNA stability, translation efficiency, and immune evasion, offering a valuable reference for studying HTLV [27,28]. Recent studies have found that N6-methyladenosine (m^6^A) modifications are enriched near the 3′ regulatory region of HTLV-1 viral RNA [29,30], but the exact modification sites, isoform specificity, and functional consequences remain undefined. Moreover, other major RNA modifications such as 5-methylcytosine (m^5^C) and pseudouridine (Ψ) have not been systematically investigated in HTLV-1. To date, no study has provided a transcriptome-wide, isoform-resolved map of RNA modifications for HTLV-1.

Taken together, although post-transcriptional regulation plays a critical role in retroviral replication, the post-transcriptional landscape of HTLV-1 remains poorly defined. At present, there is no comprehensive dataset describing full-length splice isoforms, isoform-specific poly(A) tail dynamics, or the epitranscriptomic architecture of HTLV-1. These gaps limit our understanding of how HTLV-1 RNA processing contributes to viral replication, persistence, and pathogenesis. Therefore, a high-resolution, long-read, transcriptome-wide characterization of HTLV-1 RNA processing is urgently needed. To thoroughly characterize the post-transcriptional processing and modification status of HTLV-1, we applied Nanopore Direct RNA sequencing (DRS) technology to capture native long-read viral transcriptomic features [31,32] and subsequently explored the transcriptome of HTLV-1 in MT2 cells comprehensively, which integrates HTLV-1 complete proviral sequences reported previously [33,34]. Compared to previously used next-generation sequencing (NGS), DRS enables direct sequencing of single-stranded long RNA chains and native RNA without requiring PCR amplification or other intermediate steps [35,36]. The results of this study provide a relative overall detection of HTLV-1 transcriptome in MT2 cells. The preliminary analyses of RNA alternative splicing, polyadenylation, and RNA epitranscriptomic modifications provide a panoramic view of HTLV-1 RNA modifications and splicing profiles and provide further support for research into the post-transcriptional processing and modification regulatory mechanisms of retroviruses.

## 2. Materials and Methods

### 2.1. Cell Culture

MT2 cells [American Type Culture Collection (ATCC)], maintained in our laboratory, were used in this study. Cells were cultured in RPMI-1640 medium (Gibco, Thermo Fisher Scientific, Waltham, MA, USA) supplemented with 10% fetal bovine serum (FBS), 100 U/mL penicillin, and 100 µg/mL streptomycin. Cells were incubated at 37 °C in a humidified atmosphere containing 5% CO_2_. Subsequent experiments were conducted when the cell density reached ≥1 × 10^6^ cells/mL and cell viability reached ≥99%.

### 2.2. RNA Extraction

Total RNA was extracted from 1 × 10^7^ MT2 cells using the RNA Extraction Kit (TaKaRa, Takara Bio Inc., Kusatsu, Shiga, Japan, 9767). In brief, a number of 10 million MT2 cells were lysed with lysate buffer, and the mixture was transferred to the gDNA Eraser Spin Column to remove impurities and gDNA. An equal volume of 70% ethanol was added to the filtrate, and the mixture was transferred to the RNA Spin Column to bind the RNA. The RNA Spin Column was cleaned with Buffer RWA and Buffer RWB. The RNA was finally eluted with 100 µL of RNase-Free dH_2_O. This total RNA preparation includes both host-derived viral transcripts and a minor fraction of progeny viral RNA from virion production in MT2 cells. HTLV-1-infected T cells have been shown to produce very few free virions in vitro, indicating that progeny RNA had a negligible contribution to the epitranscriptomic dataset [37]. NanoDrop™ One Microvolume UV-Vis Spectrophotometer (Thermo Fisher, Waltham, MA, USA) was used to detect the concentration and purity of the extracted RNA. Ratios of A260/A280 in the range of 1.8–2.0 and A260/A230 in the range of 1.8–2.0 were considered indicative of high-purity RNA and could be used for the next step.

### 2.3. In Vitro Transcription of RNA

The DNA templates for in vitro transcription (IVT) were prepared by RT-PCR using the PrimeScript™ One Step RT-PCR Kit Ver.2 (Takara, RR055A), with six pairs of HTLV-specific primers containing the T7 promoter sequence and poly(T) tail (Table S1). The total RNA extracted from MT2 cells was used as PCR template. DNA products were purified and recovered using a gel extraction kit (Wizard^®^ SV Gel and PCR Clean-Up System, Promega, Madison, WI, USA, A9282). DNA concentration and purity were assessed using a NanoDrop™ One Microvolume UV-Vis Spectrophotometer (Thermo Fisher), and DNA in the range of 1.8–2.0 for A260/A280 and 1.8–2.0 for A260/A230 was considered as eligible DNA. Purified DNA fragments were sequenced to confirm the accuracy of the amplified sequences. RNA was synthesized in vitro using the T7 High Yield RNA Transcription Kit (Vazyme, Nanjing, China, TR101) with the PCR-purified product as the template. The resulting in vitro transcription product was then purified using the Monarch^®^ Spin RNA Cleanup Kit (NEB, Ipswich, MA, USA, T2050L).

### 2.4. Nanopore Direct RNA Sequencing

The DRS library was prepared using the SQK-RNA004 Kit (Oxford Nanopore, Oxford, UK, SQK-RNA004). A total of 1 μg of total RNA was mixed with reverse transcription adapter, NEBNext Quick Ligation Reaction Buffer (NEB, B6058), and T4 DNA ligase (NEB, M0202T/M), and incubated at room temperature for 50 min. A reverse transcription mix containing dNTPs, 5 × reverse transcription buffer, and SuperScript™ III Reverse Transcriptase (Thermo Fisher, 18080044) was then added. The mixture was incubated at 50 °C for 50 min, followed by 70 °C for 10 min to terminate the reaction. The resulting RNA-cDNA hybrid was purified using Agencourt RNAClean XP magnetic beads (Beckman, CA, USA, A63987). A second ligation was performed with the RNA ligation adapter, T4 DNA ligase, and ligation buffer, followed by bead purification and elution in RNA Elution Buffer. The final library concentration was determined using Qubit™ RNA HS Assay Kit (Thermo Fisher, Q32852). Before sequencing, the FLO-MIN004RA RNA flow cell (Oxford Nanopore, FLO-MIN004RA) was primed using a mixture of RNA Flush Tether and Flow Cell Flush to prepare the nanopores. The library was prepared for loading by mixing 12 μL of RNA library with 25.5 μL of Library Solution (LIS) and 37.5 μL of Sequencing Buffer. The RNA library was added dropwise to the SpotON sample port. Sequencing was then carried out on the MinION platform using an R10.4.1 flow cell (Oxford Nanopore, Oxford, UK) for 72 h.

### 2.5. Bioinformatics Analysis

Basecalling was performed using Dorado (v0.8.1) [38] with the rna004_130bps_sup@v5.1.0 model, aligning reads to the HTLV reference genome (GenBank: AF003887.1) via the --reference option. The mapped sequences were sorted and indexed using Samtools (v1.2.1) [39]. Reads with mapping quality scores < 10 were excluded from downstream analysis using the --q 10 option. Read quality and length distributions were assessed using NanoPlot (v1.42.0) [40]. Alternative splicing events were quantified using Megadepth (v1.2.0) [41], and only canonical GT–AG splice junctions were annotated when splicing sites were unknown. Subsequently, we used Samtools to extract the poly(A) tail data output by Dorado. In addition, Modkit (v0.5.0) [42] was used to extract RNA modification signals. Differential methylation analysis between the sample and IVT control data was conducted using the --dmr parameter. Modification calling was performed using Modkit with the --extract parameter, extracting base modifications from individual reads, followed by integration and stratification based on transcript type.

To mitigate biases in transcript analysis arising from HTLV proviral integration, we calibrated our results using HTLV proviral DNA sequencing data from the public NCBI SRA database (PRJNA520252). Specifically, we constructed an HTLV proviral consensus sequence from 98 SRR DNA-seq runs using BWA (v0.7.19) [43], followed by mapping the DRS RNA-seq data onto this consensus with Minimap2 (v2.28) [44] and extracting splicing junctions via Regtools (v1.0.0) [45] to assess novel splicing rates and variant overlaps.

## 3. Results

### 3.1. Overview of HTLV Sequencing and Data Quality Assessment

**Figure 1:**
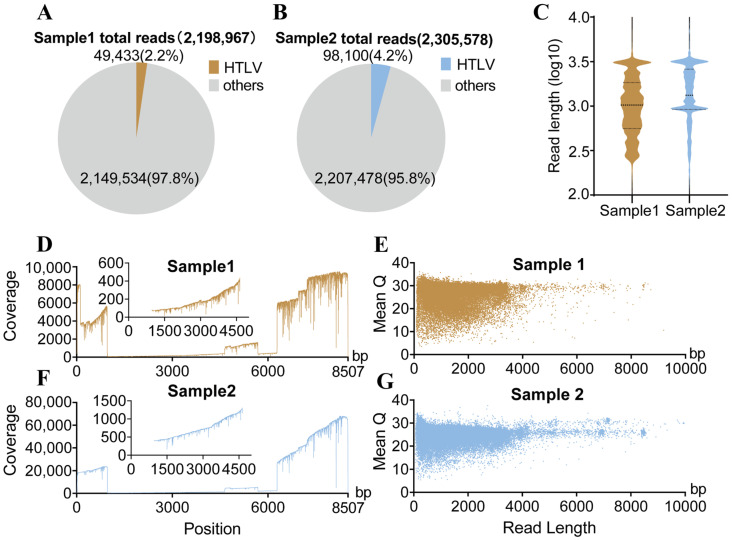
The statistics and features of ONT direct RNA sequencing data from MT2 cells. (**A**) Proportion of HTLV reads in sample 1. (**B**) Proportion of HTLV reads in sample 2. (**C**) Distribution of read lengths for sample 1 and sample 2. (**D**) Genome coverage of sequencing data from sample 1. (**E**) Relationship between sequence length and average Q values in sample 1. (**F**) Genome coverage of sequencing data from sample 2. (**G**) Relationship between sequence length and average Q values in sample 2.

To gain insight into the original features of HTLV transcriptome in MT2 cells, we extracted total RNA from MT2 cells, and DRS was then performed. MT2 cells cultured under the same culture conditions in different batches were subjected to two DRS runs as biological replicates (Samples 1 and 2). As a result, a total of 2,198,967 high-quality reads were obtained for sample 1 and 2,305,578 reads for sample 2. Among them, HTLV-related sequences accounted for 2.2% and 4.2% of the total reads, respectively (Figure 1A,B). Although the sequencing data covered the entire HTLV genome, the coverage distribution was notably uneven: In sample 1, the 5′ and 3′ ends reached a sequencing depth of over 6000, peaking at 10,000, whereas the middle region achieved only approximately 300. In sample 2, the coverage at both ends reached over 40,000, peaking at 80,000, while the central region showed only approximately 1000 (Figure 1D,F). However, the 5′ and 3′ terminal regions exhibited significantly higher coverage than the middle portion of the genome in both samples. This pronounced disparity in sequencing depth was likely attributable to the alternative splicing pattern of HTLV. Despite the observed variation in depth, both samples demonstrated consistent distributions in read length and Q-score. Most of the reads for sample 1 and sample 2 were in the 1000–3000 range (Figure 1C). The average Q-scores for sample 1 and sample 2 were above 20. Q-scores generally increased with read length, and most long reads had Q-scores above 25, indicating high-quality long-read data. Only a small proportion of long reads showed fluctuations in sequencing accuracy (Figure 1E,G). Overall, despite regional differences in sequencing depth, the sequencing quality was sufficient to provide a reliable foundation for downstream analysis of HTLV transcripts. In the subsequent analysis, paired *t*-test analysis was performed using sample 1 as a reference, yielding *p* > 0.05 across all features and thereby supporting the reproducibility and stability of our analyses.

### 3.2. Alternative Splicing Analysis of HTLV-1 Transcripts

**Figure 2:**
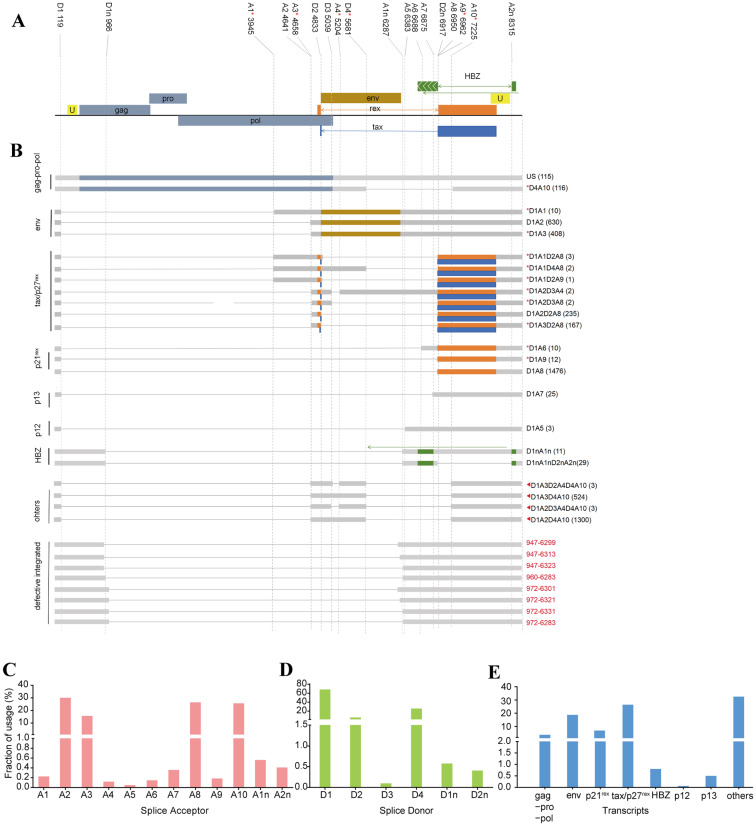
Transcript expression patterns and splice site usage of HTLV-1. (**A**) Schematic representation of splice donor and acceptor sites mapped across the HTLV-1 genome. (**B**) Classification of major HTLV-1 transcript isoforms. Each transcript is shown with respect to splice donor and acceptor usage. The numbers in parentheses indicate the read counts supporting each transcript. Newly identified splice sites and transcripts utilizing these sites are marked with a red asterisk (*). Red triangles (◀) indicate novel transcripts that do not fully cover any known coding sequence regions of HTLV-1. Transcripts shown in red font represent HTLV-1 sequences integrated into the MT2 cellular genome. (**C**,**D**) Usage frequencies of splice acceptor sites across the HTLV-1 genome. (**E**) Usage frequencies of splice donor sites.

Based on the DRS data of MT2 cells, the alternative splicing analysis of the HTLV transcripts was performed and revealed considerable HTLV-related transcriptomic diversity. As a result, certain HTLV transcripts retained multiple exons and formed diverse isoforms through various splice site combinations. A total of 18 splice sites were detected, among which were 6 donor sites and 12 acceptor sites (Figure 2A). After statistical analysis of the frequency of donor site usage, it was found that D1 (67.5%) was the most commonly used donor site, followed by D4 (25.6%). As for the usage of splicing acceptor sites, A2 (30.4%) was found to be the most commonly used site, followed by A8 (26.4%), A10 (25.6%) and A13 (15.4%). The remaining acceptor sites had much lower usage frequencies (Figure 2C,D). Despite the 12 reported splicing sites (5 donor sites and 7 acceptor sites), we discovered 6 new splicing sites that have not been reported before, among which were 1 donor site and 5 acceptor sites. Among these sites, only A3 is used frequently, while the other sites are used less often, indicating that the transcripts relied on them may only serve as supplements.

To assess whether HTLV-1 transcripts in MT2 cells originate from proviral integration, we mapped DRS reads onto an HTLV-1 proviral consensus constructed from NCBI SRA (PRJNA520252). Only a very small number of splicing methods (*n* = 20) corresponded to the consensus, whereas the vast majority (*n* = 919) did not match the proviral reference, indicating that these RNAs do not arise from incomplete proviral integration but instead reflect transcription from the viral genome and its variable splicing events (Figure S2 and Table S2).

Furthermore, we identified multiple RNA isoforms resulting from the selection of different splice donor and acceptor sites (Figure 2B). A total of 23 RNA isoforms were detected, including 8 known isoforms and 15 new ones [14,15]. Among the novel isoforms, 9 represented previously reported defective HTLV-1 integrations in the MT2 genome [34]. Among the known isoforms, those associated with tax/p27^rex^ were the most abundant, while p13 and p12 isoforms were the least. In terms of expression levels, the p21^rex^ transcript had the highest expression, followed by env and tax/p27^rex^ (Figure 2E). Additionally, we detected a group of RNA transcripts (others) that did not correspond to any annotated HTLV isoforms. These variants are unannotated in the reference genome annotations, lacking described functional roles, but all shared a common and highly conserved splice donor site D1, which was consistently present in known HTLV isoforms and showed strong consistency across both samples. Although the functions of these unannotated transcripts remain unclear, the conservation of D1 suggests they are genuine alternative splicing products rather than sequencing artifacts or random transcriptional noise. Furthermore, a novel splicing form, D4A10, spanning the gag, pro, and pol regions, is classified as a gag-pro-pol transcript even though prior studies suggest this class typically arises from unspliced transcripts [14]. Our discovery of numerous novel transcript isoforms underscores the intricate post-transcriptional regulation of HTLV-1.

In summary, HTLV RNA exhibited complex alternative splicing patterns, diverse isoform types, and notable splice site usage bias. The presence of defective integrated sequences suggests that HTLV may engage in regulatory interactions with the host genome, possibly contributing to as-yet-unclear functional roles in viral gene expression.

### 3.3. Polyadenylation Analysis of HTLV-1 Transcripts

**Figure 3:**
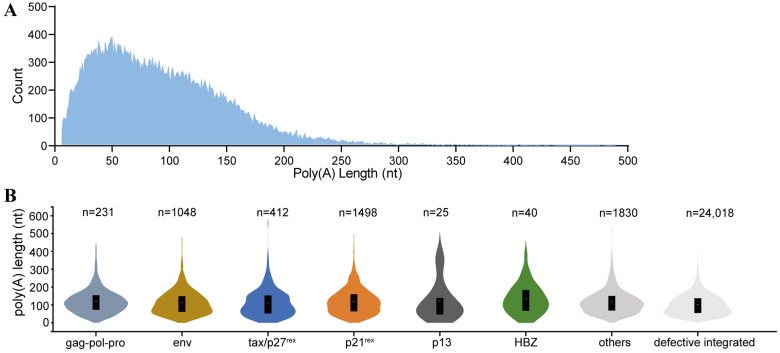
Poly(A) tail length distribution of HTLV-1 transcripts. (**A**) Global distribution of poly(A) tail lengths for all HTLV-1 transcripts. (**B**) Distribution of poly(A) tail lengths for each transcript.

Polyadenylation is an important component of RNA modification, and poly(A) tails play a crucial role in maintaining mRNA stability and promoting mRNA translation. In this study, we detected the poly(A) tail length of HTLV transcripts in MT2 cells and analyzed the relationship between polyadenylation and mRNA expression (Figure 3 and Table 1).

As shown in Figure 3A, the poly(A) tail lengths of most transcripts were distributed within the 0–200 nt range, with a prominent peak around 50 nt. Transcripts with poly(A) tails longer than 200 nt, particularly those exceeding 300 nt, were extremely rare. At the individual transcript level, tax/p27^rex^ had the longest average tail (131 nt), followed by gag-pro-pol (129 nt), p21^rex^ (127 nt), p13 (117 nt), env (112 nt), and HBZ (108 nt). There were clear differences in poly(A) tail length distributions among the various transcripts, and the overall distribution showed a statistically significant difference (*p* < 0.05). Note that p12 was not included in the statistics due to the small quantity (*n* = 3) (Figure 3B).

Overall, distinct differences in poly(A) tail lengths were observed among different HTLV transcripts in MT2 cells. This may reflect the complexity of poly(A) tail regulation, differences in transcript abundance, or inherent variability in retroviral polyadenylation mechanisms.

### 3.4. Epi-Transcriptome Analysis of HTLV-1

**Figure 4:**
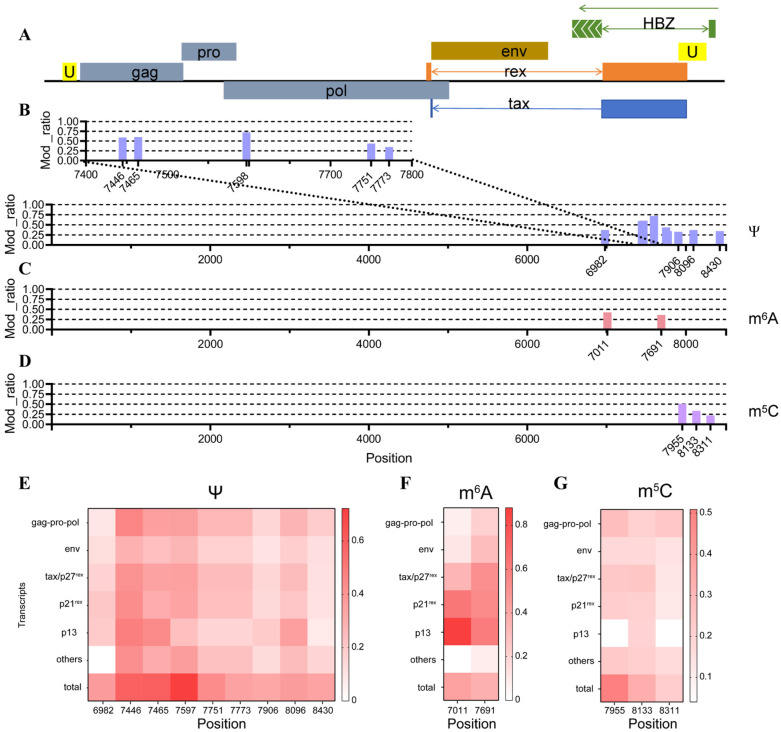
RNA modification profiles of HTLV-1 transcripts. (**A**) Schematic representation of the HTLV-1 genomic structure. (**B**–**D**) Modification sites and modification rates of pseudouridine (Ψ), N^6^-methyladenosine (m^6^A), and 5-methylcytidine (m^5^C). (**E**–**G**) Modification rates of Ψ, m^6^A, and m^5^C across different transcript categories.

RNA epitranscriptomic modification is an important regulatory mechanism for viral replication. To investigate the epitranscriptomic features of HTLV transcripts, we conducted modification prediction using Dorado. Three types of RNA modifications were analyzed: N^6^-methyladenosine (m^6^A), 5-methylcytosine (m^5^C), and pseudouridine (Ψ). To reduce background noise and false positives, we included in vitro transcribed (IVT) RNA lacking modifications as a negative control (Figure S1). We used the thresholds of valid_coverage > 1000 and percent_modified > 20%. Following the literature and our data, a total of 14 high-confidence modification sites were identified: 9 (Ψ) sites, 2 m^6^A sites, and 3 m^5^C sites (Figure 4A–D). These modification sites were predominantly concentrated near the 3′ end of the transcripts. The identified Ψ sites were located at positions 6982, 7446, 7465, 7598, 7751, 7773, 7906, 8096, and 8430, with the highest percent_modified at position 7598 (0.72). The m^6^A sites were identified at positions 7011 and 7691, both with percent_modified values around 0.4. The three m^5^C sites were located at positions 7955, 8133, and 8311, while site 7955 showed the highest modification level (0.50).

In order to detect the different modification rates of HTLV-1 transcripts reported before, we analyzed the modification type and mod_ratio at each site of every HTLV-1 transcript separately (Figure 4E–G), excluding low-abundance transcripts as previously noted. As a result, the modification ratios in these transcripts were generally lower than the overall levels. For the 9 pseudouridine modification sites, all transcripts exhibited mod_ratios lower than the overall level (Figure 4E). The m5C mod_ratio across all transcripts was consistently lower than the global level, mostly around 20% (Figure 4G). For m6A modifications, the modification ratio at site 7691 in the tax/p27^rex^ transcript exceeded the global level, reaching up to 51% (Figure 4F). In p21^rex^ and p13 transcripts, both modified sites exhibited higher mod_ratio than the overall average, while in other transcripts, the ratios for the two sites mostly centered around 15%. When grouped by transcript type, most isoforms displayed modification rates lower than the global average, with the exception of p13, which showed higher m^6^A modification levels. These high-confidence modification sites showed clear positional enrichment, suggesting that HTLV may employ RNA chemical modifications to regulate the expression or function of specific transcript regions.

In conclusion, this study provided a comprehensive overview of the structural complexity and chemical modifications of HTLV transcripts and revealed extensive alternative splicing, variations in poly(A) tail length, and region-specific RNA modifications, respectively. These results collectively offered foundational insights for future investigations into HTLV gene regulation and virus–host interactions.

## 4. Discussion

HTLV-1, as the first human retrovirus to be identified, provides important insights into the understanding of other retroviruses [5]. Although previous studies have analyzed the genomic structure and some aspects of the epitranscriptome of HTLV-1, research on RNA modifications, alternative splicing isoforms, and poly(A) tail length remains relatively limited [12,29,30]. Notably, nanopore DRS technology allows the direct detection of transcriptome without reverse transcription and amplification [31,32]. The sequence data can give messages of RNA modifications, splicing events and polyadenylation at the single-molecule level, offering unprecedented advantages.

In this study, we used DRS to profile the HTLV-1 post-transcriptional processing and modification thoroughly. For splicing events of HTLV-1, we identified 23 RNA isoforms, including canonical HTLV-1 transcripts consistent with previous reports as well as defective integrated transcripts in MT2 cells, reflecting major transcriptional patterns in this cellular context [34]. Notably, while many typical transcripts were captured, we failed to detect the p30-related splice product, consistent with its low and variable expression in HTLV-1-infected cell lines [46]. Our data revealed fragmented integrated HTLV-1 transcripts, providing further evidence for the integration of HTLV-1 in MT2 cells [15,34]. We characterized multiple isoforms of the HTLV-1 RNA nuclear export factors tax/rex [47], which can promote viral replication and cellular senescence [48]. This high expression is reflected in clinical settings where the total amount of HTLV-1 tax mRNA in peripheral blood mononuclear cells was significantly higher in HAM/TSP patients than in asymptomatic carriers and correlated with proviral load and disease severity, linking to aggressive progression in HTLV-1-associated neuroinflammation [49]. These isoform discoveries highlight the complexity of tax/rex’s cellular effects during infection, providing new insights for ATL and HTLV-1-associated myelopathy/tropical spastic paraparesis. Besides this, numerous viral transcripts with no research reports and no detailed functional descriptions in the annotation file information have been discovered as well. This phenomenon gives insight into the regulation of retroviral genome, underscoring substantial variability in HTLV-1 transcript processing; such variations could potentially influence translation efficiency, protein structure, or RNA stability, offering new entry points to investigate HTLV-1 transcriptional regulation [50,51]. While these novel isoforms suggest potential regulatory parallels in retroviral epitranscriptomes, functional validation remains essential. These findings offer a solid foundation for further elucidating the roles of alternative splicing in HTLV-1 viral replication and host immune evasion, while supporting advanced investigations into ATL and HAM/TSP therapeutics [52,53,54].

Previous studies have shown that the 3′ end polyadenylation plays a crucial role in translation efficiency and RNA stability in different organisms [22,23,55,56]. A former study of our research group revealed that the poly(A) tail of HIV-1 subtype B (NL4-3) transcripts exhibits complex length distribution but concentrates around 50–100 nt [57]. In this study, the poly(A) tail length of HTLV-1 transcripts was found to concentrate around 50–100 nt. In host cells, poly(A) tails are synthesized in the nucleus to defined lengths (approximately 250 nt in mammals) and subsequently shortened in the cytoplasm at transcript-specific rates, leading to steady-state tail length distributions maintained by a balance between polyadenylation and deadenylation activities [58]. Although this mechanism has been characterized primarily in cellular mRNAs, the approximately 50–100 nt tail length observed for HTLV-1 likely reflects regulated steady-state control rather than the initial nuclear synthesis length. Furthermore, our analysis of the data revealed differences in poly(A) lengths between different transcripts, and the differences were statistically significant, suggesting that the regulatory mechanism of poly(A) tail length on transcript translation is complex and precise. These findings suggest that, despite being generally constrained within a stable range, HTLV-1 poly(A) tails may mediate isoform-specific regulatory functions, with implications for isoform-dependent regulation in the viral life cycle.

RNA modifications can enhance protein translation and RNA stability, promote nuclear export, and suppress innate immune recognition [59]. Most functional studies on retroviral modifications have focused on HIV, where, for example, m^6^A has been shown to promote translation of Gag proteins, strengthen Rev–RRE interactions, and enhance nuclear export; m^5^C contributes to RNA stability and translation; and Ψ influences RNA secondary structure, thereby regulating splicing and translation [27]. In contrast, investigations on HTLV-1 RNA modifications remain scarce. Only recently was it reported that m^6^A in HTLV-1 is enriched at the 3′ end, with site-specific mapping still limited [29,30]. To address this gap, we used DRS to systematically identify RNA modification sites in HTLV-1 transcripts from MT2 cells. By comparing the raw electrical current signals from our cellular HTLV-1 RNA against this unmodified IVT baseline, we were able to confidently identify sites with altered current patterns indicative of modifications, effectively excluding false positives caused by sequence motifs or basecalling errors [60]. Our results were consistent with earlier observations [29]: m^5^C, m^6^A, and Ψ modifications were mostly enriched near the 3′ end of HTLV-1 RNA, a pattern similar to the modification distribution trend observed in HIV-1 [60]. Existing studies have shown that there are many similarities in the post-transcriptional processing of HTLV-1 and HIV-1 [47,61], and our research further supports this resemblance. This convergence likely stems from conserved transcription and replication strategies among retroviruses, such as enhanced 3′ UTR stability that facilitates viral genome packaging and integration into host cells [62]. Additionally, modifications at the 3′ end may also influence the nuclear export and translation efficiency of viral mRNA, potentially contributing to viral replication and immune evasion [63]. Thus, the enrichment of 3′ end modifications could be a common feature of retroviruses, and its specific role in the viral lifecycle warrants further investigation. Furthermore, the overall modification rates at individual sites were generally higher than those at the transcript level, potentially due to the potential impact of MT2 cell integration site biases. MT2 cells contain numerous defective HTLV-1 proviral integrations, whose RNA transcripts outnumber those from intact proviruses, thereby lowering site-specific modification rates at the transcript level compared to the global average [34]. Taken together, our study shows that the distribution pattern of HTLV-1 modifications resembles that reported for HIV-1. This resemblance advances understanding of HTLV-1 virology and, by comparison, offers perspective on shared molecular mechanisms among retroviruses. Given the commonalities between the two viruses in replication cycle and host interactions, findings from HTLV-1 studies may, to some extent, inform broader approaches to understanding and targeting retroviral infections. However, although MT2 cells are ideal for generating high-abundance viral RNA requisite for initial DRS profiling, they may not fully capture the heterogeneity present in primary patient samples, such as those from ATL or HAM/TSP. Therefore, our findings should be viewed as a high-resolution atlas within the context of MT2 cells, serving as a foundational resource to guide future comparative investigations in more physiologically relevant models.

Together, our characterization of HTLV-1 alternative splicing isoforms, 3′ end polyadenylation profiles, and RNA modification landscapes provides novel insights into the post-transcriptional regulation of this virus. The observed similarities in poly(A) tail length distribution and 3′-end-enriched RNA modifications between HTLV-1 and HIV-1 point to potentially conserved strategies adopted by distinct retroviruses to fine-tune RNA stability, translation, and replication efficiency. These parallels highlight HTLV-1 as a valuable comparative model for dissecting shared and virus-specific regulatory mechanisms within the Reoviridae family. Future studies integrating functional assays with high-resolution transcriptomic profiling across multiple retroviruses will be essential to clarify whether these features represent universal RNA regulatory signatures and to explore their potential as novel targets for broad-spectrum antiviral interventions.

## Figures and Tables

**Table 1:** Mean poly(A) length of transcripts of different splicing forms of HTLV-1.

Transcripts	Splice Pattern	Mean Poly(A) Length
gag-pro-pol	Unspliced (115)	140
	D4A10 (116)	110
env	D1A1 (10)	164
	D1A2 (630)	112
	D1A3 (408)	112
tax/p27^rex^	D1A1D2A8 (3)	269
	D1A1D4A8 (2)	98
	D1A1D2A9 (1)	63
	D1A2D3A4 (2)	121
	D1A2D3A8 (2)	221
	D1A2D2A8 (235)	102
	D1A3D2A8 (167)	168
p21^rex^	D1A6 (10)	117
	D1A9 (12)	127
	D1A8 (1476)	127
p13	D1A7 (25)	117
p12	D1A5 (3)	125
HBZ	D1nA1n (11)	100
	D1nA1nD2nA2n (29)	130
others	D1A3D2A4D4A10 (3)	67
	D1A3D4A10 (524)	119
	D1A2D3A4D4A10 (3)	178
	D1A2D4A10 (1300)	113
defective integrated	947-6299 (5)	106
	947-6313 (8)	84
	947-6323 (30)	104
	960-6283 (23,678)	102
	972-6301 (4)	43
	972-6321 (14)	80
	972-6331 (95)	91
	972-6283 (184)	103

## Data Availability

Raw data is available at National Center for Biotechnology Information under https://www.ncbi.nlm.nih.gov/bioproject/1335625 (accessed on 20 December 2025).

## Supplementary Materials

The following supporting information can be downloaded at https://www.mdpi.com/article/10.3390/v18010057/s1, Figure S1: The statistics and features of ONT direct IVT sequencing data from MT2 cells; Figure S2: The pie chart of mapping of MT2 direct RNA-seq reads to the HTLV-1 proviral consensus constructed from PRJNA520252; Table S1: Primers Used for In Vitro Transcription; Table S2: Mapping of MT2 direct RNA-seq reads to the HTLV-1 proviral consensus constructed from PRJNA520252.

## Abbreviations

The following abbreviations are used in this manuscript:

HTLV-1	Human T-lymphotropic virus type 1
ALT	adult T-cell leukemia
HAM/TSP	myelopathy/tropical spastic paraparesis
m^6^A	N6-methyladenosine
m^5^C	5-methylcytosine
Ψ	pseu-douridine
DRS	direct RNA sequencing
