# Supplementary material for: Transcriptomic and Epitranscriptomic Landscape of Integrated HTLV-1 in MT2 Cells
Source: Viruses. 2025 Dec 30;18(1):57. doi: 10.3390/v18010057 (PMC12846610; doi:10.3390/v18010057)
Supplement: Supplementary file 1 [file viruses-18-00057-s001.zip › figure S1.pdf]

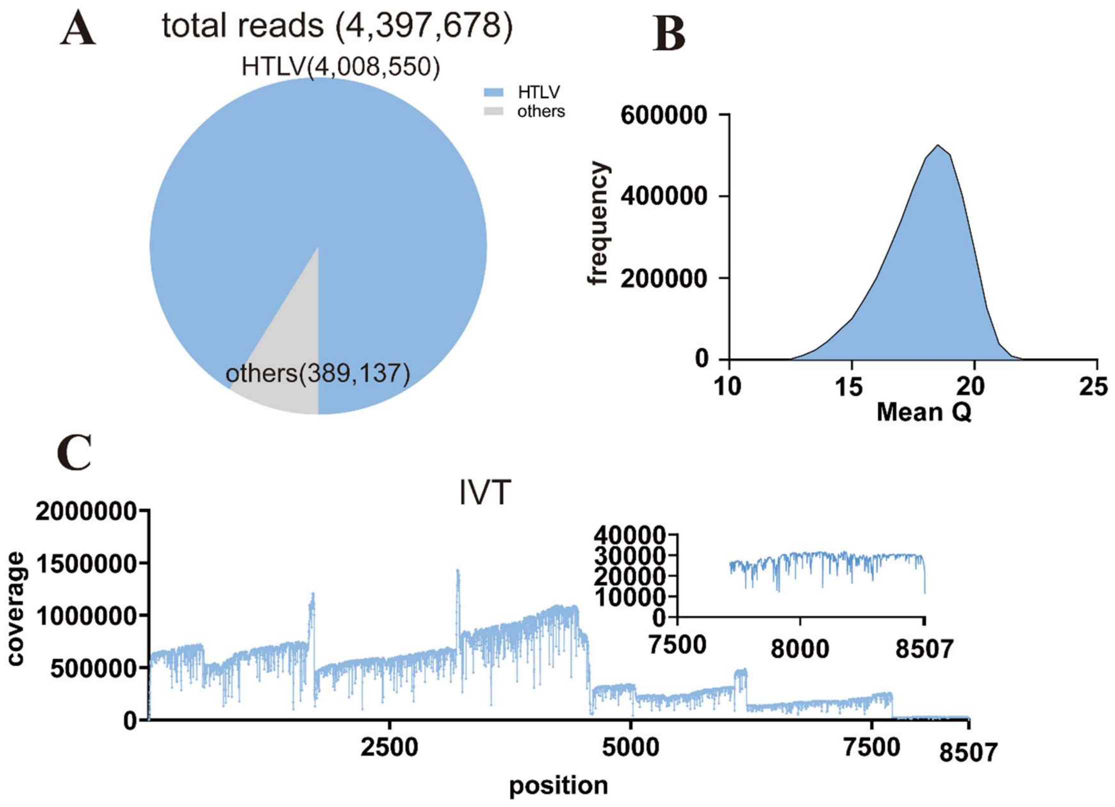

Figure S1. The statistics and features of ONT direct IVT sequencing data from MT2 cells. (A) Proportion of HTLV reads in sample1. (B) Distribution of read lengths for IVT. (C) Genome coverage of sequencing data from IVT.
