# Supplementary material for: Transcriptomic and Epitranscriptomic Landscape of Integrated HTLV-1 in MT2 Cells
Source: Viruses. 2025 Dec 30;18(1):57. doi: 10.3390/v18010057 (PMC12846610; doi:10.3390/v18010057)
Supplement: Supplementary file 1 [file viruses-18-00057-s001.zip › figure S2.pdf]

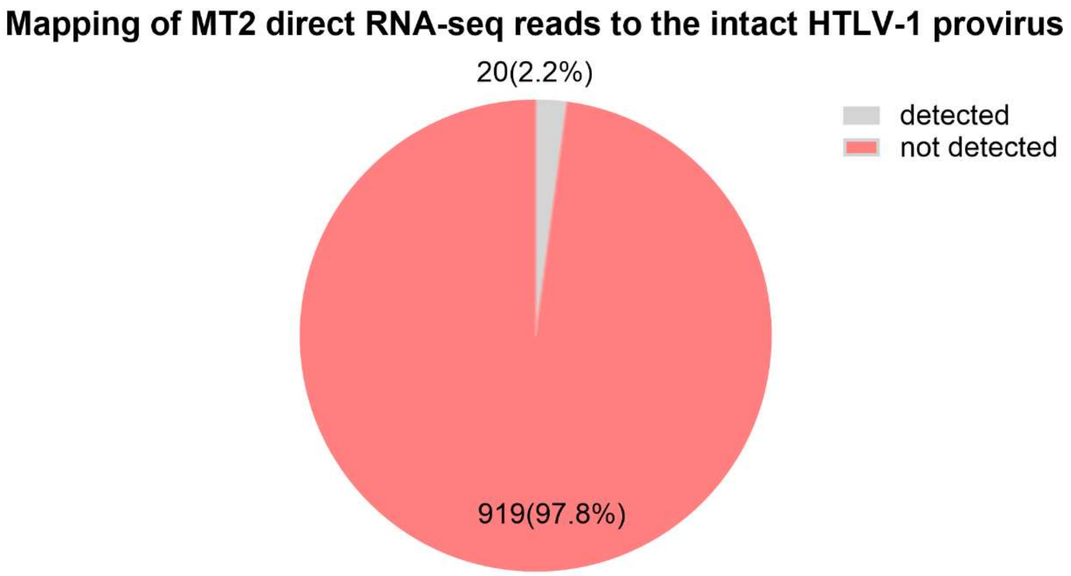

Figure S2. The pie chart of mapping of MT2 direct RNA-seq reads to the HTLV-1 proviral consensus constructed from PRJNA520252. Gray sectors representing detected reads (aligned to the constructed consensus) and red sectors representing not detected reads (unaligned to the constructed consensus).
