# Supplementary material for: Transcriptomic and Epitranscriptomic Landscape of Integrated HTLV-1 in MT2 Cells
Source: Viruses. 2025 Dec 30;18(1):57. doi: 10.3390/v18010057 (PMC12846610; doi:10.3390/v18010057)
Supplement: Supplementary file 1 [file viruses-18-00057-s001.zip › Table S1.pdf]

Table S1. Primers Used for In Vitro Transcription

Primer Name	Primer Sequence (5′-3′)
HTLV-IVT-1-F	CCCCTAATACGACTCACTATAGGGGGCTCGCATCTCTCCTTCAC
HTLV-IVT-1-R	TTTTTTTTTTTTTTTTTTTTTTTTTTTGGGTGTGGGATGTCG
HTLV-IVT-2-F	CCCCTAATACGACTCACTATAGGGAACCAGAGCCAGAGGAGGAT
HTLV-IVT-2-R	TTTTTTTTTTTTTTTTTTTTTTGGGCATGTAGCCAGACAAGT
HTLV-IVT-3-F	CCCCTAATACGACTCACTATAGGGCTATTATGCTGACCCTCACTGG
HTLV-IVT-3-R	TTTTTTTTTTTTTTTTTTTTTTGCTTCTTGAAGAGCCTCCTG
HTLV-IVT-4-F	CCCCTAATACGACTCACTATAGGGCCCTCAGCAATAAACAAACC
HTLV-IVT-4-R	TTTTTTTTTTTTTTTTTTTTTTCAAGGATAACAAGAAGGAGTAGC
HTLV-IVT-5-F	CCCCTAATACGACTCACTATAGGGAAGAGAGACCCCCCCTTGAA
HTLV-IVT-5-R	TTTTTTTTTTTTTTTTTTTTTATAGGCGTGCCATCGGTAAA
HTLV-IVT-6-F	CCCCTAATACGACTCACTATAGGGTTCCCTACAAACGAATAGAAAAACT
HTLV-IVT-6-R	TTTTTTTTTTTTTTTTTTTTTAAGAGTTGAGCAAGCAGGGTC
