# Supplementary material for: Transcriptomic and Epitranscriptomic Landscape of Integrated HTLV-1 in MT2 Cells
Source: Viruses. 2025 Dec 30;18(1):57. doi: 10.3390/v18010057 (PMC12846610; doi:10.3390/v18010057)
Supplement: Supplementary file 1 [file viruses-18-00057-s001.zip › Table S2.pdf]

Table S2. Mapping of MT2 direct RNA-seq reads to the HTLV-1 proviral consensus constructed from PRJNA520252

Strat	End	chrom
1	5546	JUNC00000003
3	5585	JUNC00000002
6	8051	JUNC00000011
17	6470	JUNC00000001
439	6281	JUNC00000025
607	6536	JUNC00000039
608	6282	JUNC00000098
661	6662	JUNC00000052
667	6467	JUNC00000028
676	6510	JUNC00000089
759	6291	JUNC00000362
800	6490	JUNC00000027
800	6662	JUNC00000014
801	6468	JUNC00000012
837	6463	JUNC00000009
965	6514	JUNC00000033
4326	7897	JUNC00000013
4912	8007	JUNC00000016
5302	7325	JUNC00000073
5317	7376	JUNC00000122
